# Disentangling Puzzles of Spatial Scales and Participation in Environmental Governance—The Case of Governance Re-scaling Through the European Water Framework Directive

**DOI:** 10.1007/s00267-016-0753-8

**Published:** 2016-09-20

**Authors:** Jens Newig, Daniel Schulz, Nicolas W. Jager

**Affiliations:** Research Group on Governance, Participation and Sustainability, Leuphana University of Lüneburg, Scharnhorststrasse 1, 21335 Lüneburg, Germany

**Keywords:** Multi-level governance, Re-scaling, Democratic dilemma, Polycentric governance, Sustainable water resources management, Mandated participatory planning

## Abstract

This article attempts to shed new light on prevailing puzzles of spatial scales in multi-level, participatory governance as regards the democratic legitimacy and environmental effectiveness of governance systems. We focus on the governance re-scaling by the European Water Framework Directive, which introduced new governance scales (mandated river basin management) and demands consultation of citizens and encourages ‘active involvement’ of stakeholders. This allows to examine whether and how re-scaling through deliberate governance interventions impacts on democratic legitimacy and effective environmental policy delivery. To guide the enquiry, this article organizes existing—partly contradictory—claims on the relation of scale, democratic legitimacy, and environmental effectiveness into three clusters of mechanisms, integrating insights from multi-level governance, social-ecological systems, and public participation. We empirically examine Water Framework Directive implementation in a comparative case study of multi-level systems in the light of the suggested mechanisms. We compare two planning areas in Germany: North Rhine Westphalia and Lower Saxony. Findings suggest that the Water Framework Directive did have some impact on institutionalizing hydrological scales and participation. Local participation appears generally both more effective and legitimate than on higher levels, pointing to the need for yet more tailored multi-level governance approaches, depending on whether environmental knowledge or advocacy is sought. We find mixed results regarding the potential of participation to bridge spatial ‘misfits’ between ecological and administrative scales of governance, depending on the historical institutionalization of governance on ecological scales. Polycentricity, finally, appeared somewhat favorable in effectiveness terms with some distinct differences regarding polycentricity in planning vs. polycentricity in implementation.

## Introduction

Fundamental questions in governance are related to issues of scale, defined here has the spatial configuration of (multi-level) governance systems. Such spatial configurations have implications for the effectiveness and legitimacy of political outputs: While decision-making processes on smaller, more local scales allow for the representation of large parts of the community and directly correspond to their preferences, many important environmental and sustainability issues can only be tackled effectively on larger scales. More remote from the citizens, however, decision-making on larger scales tends to fall short of democratic legitimacy. This tension has been termed “democratic dilemma between system effectiveness and citizen participation” (Dahl 1994). It is of particular importance in environmental governance, where issues are typically complex, with increasing spatial connectedness, and transgressing administrative jurisdictions (Meadowcroft 2002; Young et al. 2006). In order to cope with such problems of spatial ‘misfit’ (Moss 2003; Young 2002), functionally specific governance institutions are increasingly implemented on scales that correspond to the geographic boundaries of environmental problems (Hooghe and Marks 2003). Following this trend, governance in the European Union (EU) and elsewhere is characterized by a multiplicity of vertical, horizontal and functionally specific levels of decision-making. Aiming to balance diverse aspects of legitimacy and effectiveness, such polycentric systems also tend to further increase governance complexity, leading to problems of transparency and accountability (Peters and Pierre 2005). To cope with these deficits, the EU has undertaken efforts to decentralize environmental decision-making and policy implementation (Jordan 2002), including the involvement of citizens and local interest groups. These efforts seek to make governance more effective, for example by incorporating local knowledge into decisions and by generating greater acceptance and implementation of decisions (Heinelt et al. 2002), and at the same time enhance the legitimacy of decision-making.

A prototypical example of such purposeful re-scaling is the EU Water Framework Directive (WFD). The WFD has introduced new governance scales (mandated river basin management) and demands consultation of citizens and encourages ‘active involvement’ of stakeholders in the course of its implementation (Jager et al. 2016; Kaika 2003; Newig and Koontz 2014). This ‘re-scaled’ (Moss 2004) structure of European water governance entails virtually all of the above sketched scale-related puzzles of democratic legitimacy and effective policy delivery, revolving around non-state actor participation in mandated planning as the central vehicle of WFD implementation.

While a diversity of disciplines—such as federalism (Dahl 1994; Oates 2002), social ecological systems (Berkes, Folke), or institutionalism (Ostrom, Young)—have been contributing a variety of aspects, there is still surprisingly little consolidated knowledge about how ‘scalar’ approaches relate to effective and legitimate environmental governance (Gerlak 2014; Newig and Fritsch 2009). This article contributes to the conceptual literatures on scalar and multi-level governance in that it systematically integrates the scalar puzzles by formulating precise mechanisms and discussing their empirical relevance in a comparative study of WFD implementation in Germany. In doing so, this article also contributes to the growing body of research on the governance implications of WFD implementation. We examine the triangular relations of scale, participation, and the normative dimensions of environmental governance (legitimacy and effectiveness) in order to address the following research questions: (1) To what extent does non-state actor participation on different levels of water governance impact the legitimacy and effectiveness of public decision-making? (2) To what extent does Dahl’s (1994) ‘democratic dilemma’ empirically exist in WFD-related multi-level systems? (3) What is the role of functionally specific multi-level governance arrangements (Hooghe and Marks 2003), institutionalized through river-basin management, and how can participation in such polycentric systems help overcome related problems of ‘fit’ (Moss 2003)?

The remainder of this article is organized as follows. In section 2, we lay out the conceptual framework, condensing propositions from different streams of literature into (causal) mechanisms. These serve to guide empirical research of a comparative case study of multi-level systems in the context of WFD implementation (section 3). The research design examines multiple levels from the EU to local catchment level, focusing on two distinct planning areas in Germany, the Wupper sub-basin in North Rhine Westphalia and the Hase sub-basin in Lower Saxony (LS). Empirical findings will be discussed in the light of the mechanisms (section 4), before we conclude by reflecting on the overall research approach and discuss avenues for further research (section 5).

## Theorizing on the Relation of Participation, Scales, Levels, Democratic Legitimacy and Environmental Effectiveness

In this section, we first define the key concepts of the analysis, such as scale, level, polycentricity, participation, legitimacy and effectiveness. Subsequently, we develop the analytical framework, integrating assumptions from the literature into a set of hypothesized causal mechanisms, linking scale, level, polycentricity, and participation with legitimacy and effectiveness.

### Definition of Key Concepts

Relying on conceptual insights from different strands of scholarly research, there are a number of somewhat conflicting mechanisms concerning the relationship between public participation and environmental outcomes. Generally, we assume that scales and levels of decision-making as well as different types of participation influence outcomes (Newig and Fritsch 2009).

Drawing on existing conceptualizations of scale (Cash et al. 2006; Gibson et al. 2000; Moss and Newig 2010), we distinguish between scalar dimension and scalar level. Scalar *dimension* refers to “an analytical dimension of a problem under study” (Moss and Newig 2010: 4). In the context of environmental governance, two dimensions are of particular importance, namely the biophysical (here: hydrological) and the institutional scalar dimension (Hein et al. 2006). Scalar *level* denotes the “units of analysis that are located at the same position on a scale” (Gibson et al. 2000: 218). Of particular importance to this research are the different levels of the EU multi-level governance system (e.g., EU—national—federal state—municipality), and the levels on the hydrological scale (basin—sub-basin—catchment).

In line with much current scholarship, we assess environmental governance against the criteria of democratic legitimacy and effectiveness (see e.g., Hogl et al. 2012).

Based on the policy cycle model (Easton 1965), three dimensions of *democratic legitimacy* can be distinguished (Scharpf 1997; Schmidt 2013). Participation can play a central role in achieving each of these forms of legitimacy in public decisions. Input-oriented legitimacy refers to the constitution of the (participant) decision-making body. A central criterion is representation of those with a ‘stake’ or other legitimate interest (see Fung 2006; Schmitter 2002 for detailed criteria). The legitimacy of democratic decisions rests to a large degree on the procedures employed, referred to as ‘throughput.’ Democratic processes allow the accommodation of different (often conflicting) interests, ensure transparency and monitoring by those not involved. This implies that procedures are fair and that participants have an actual say in decisions. Finally, output-oriented legitimacy, has been defined as a measure of acceptance of the output on the part of all affected parties (cf. Benz 2001).

*Effectiveness,* on the other hand, describes the substantive dimension of policy-making. Like legitimacy, the concrete measurement of effectiveness is challenging (Koontz and Thomas 2006; Newig and Fritsch 2009; Young 2011). This has to do with often complex causal chains of intermediate steps from decision-making to tangible impacts on environmental quality. To aid analysis, we draw on the literature on the effectiveness of environmental institutions (Mitchell 2008), distinguishing output, outcome and impact. To this, we add the dimension of substantive process quality. Applying this approach to the effectiveness of participatory arrangements to reach the goals of the WFD, i.e., the attainment of good ecological status, we arrive at the following criteria:

- Process quality: the extent to which participation gauges ecologically relevant information from participants that can be included in the processes of planning and developing measures for the implementation of the WFD;
- Output: the extent to which decision outputs (River Basin Management Plans (RBMP) and supplementary documents such as “implementation timetables”) align with the goals of the WFD;
- Outcome: indications of actual implementation of measures toward reaching the WFD’s goals.
- Impact: Changes (improvements or deteriorations) of water status (river structure, nutrient load, etc.) as measured by states’ and EU official reports.

Several issues arise with this measurement of effectiveness. First, we need reliable information on how information was gathered from participants during participatory processes. This has been possible in most cases. Second, decision outputs (RBMP and other documents) must be sufficiently clear in their content to allow for a comparison with WFD aims and goals. In practice, plans are often either quite general or remain vague or cryptic in the actual measures they contain. Third, implementation activities on the ground are manifold and often decentralized; our assessment via interviews and document analysis reveals indications but not necessarily a complete picture of implementation activities. Finally, it is difficult to assess the actual impact of most of the implemented measures because of the long-term nature of many of the involved biophysical processes (Koontz and Thomas 2006). Notwithstanding these methodological challenges, the diversity of indicators employed does allow for a nuanced assessment of effectiveness, including the uncertainties at stake.

### Analytical Framework for Analysis

**Fig. 1:**
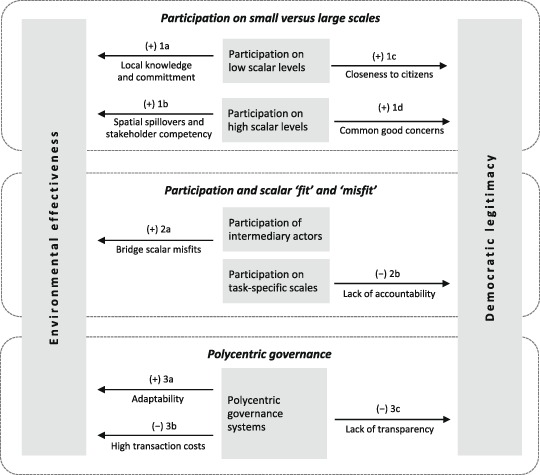
Overview of conceptual framework comprising three clusters of mechanisms, which link scale-related factors to environmental effectiveness and democratic legitimacy

In the following, we summarize what emerges from several literatures as key causal mechanisms. Following Elster (1989), we suggest that “[a] mechanism explains by opening up the black box and showing the cogs and wheels of the internal machinery. A mechanism provides a continuous and contiguous chain of causal or intentional links between the explanans and the explanandum” (cited in Hedström and Ylikoski 2010: 51). In our analysis, mechanisms link scale and participation in governance with legitimate and effective environmental decision-making. We organize these into three clusters regarding participation on small vs. large scales (1), scalar ‘fit’ (2), and polycentric governance (3). Figure 1 presents an overview of all mechanisms. It is not our intention, nor is it possible with the present research design, to ‘test’ these assumptions with any standard of rigor. Rather, we use these as a focused lens to discern relevant issues in the studied cases of WFD implementation that can be connected to and interpreted in the light of existing conceptualizations.

#### Participation and Scalar Level: Participation on Small vs. Large Scalar Levels

Environmental federalism (Oates 2002) has long debated on what level environmental decisions are most efficiently taken. Much of the participation-related literature holds local-level participation to be particularly suited to reaching effective decisions (Bingham 1986). Citizens and stakeholders, including environmental non-governmental organizations (NGOs), living in close proximity to the relevant environmental resources are assumed to often possess a more detailed, comprehensive, and contextualized understanding of these resources than do the responsible authorities (Steele 2001; Thomas 1995). Local actor participation is thus expected to lead to better informed decisions (Pellizzoni 2003; Yearley et al. 2003). Other scholars stress the importance of social cohesion and the construction of social capital at local levels fostering trust, commitment and ownership among participants and, hence, contributing to a common problem-solving capacity (Cheng and Daniels 2003; Newig and Fritsch 2009). Moreover, solutions developed and rooted in such a socially cohesive and committed environment are expected to more likely generate high levels of implementation and greater compliance (Ostrom 1990). Taken together, these arguments constitute

Mechanism 1a: Participatory governance on low scalar levels is conducive to environmental effectiveness.

Opposing this mechanism, and drawing again on Dahl (1994), there are strong arguments to support that collective matters with regard to environmental problems can typically be dealt with more effectively on wider (e.g. national or supranational) rather on than very local scalar levels (Flynn 2000). Given negative environmental spillovers (Benson and Jordan 2010) of local activities, attempts to solve such problems locally represent a collective-action dilemma (Hardin 1968; Olson 1969). Moving to higher spatial levels of decision-making can internalize such spillovers, making pro-environmental decisions more likely. Moreover, local administration is assumed to be more susceptible to lobbying (regulatory capture) by economic development interests or—in a more favorable light—interested to negotiate local exceptions from stricter national legislation (van Stigt et al. 2016). In addition, participants at higher levels of governance are assumed to possess greater professional competency (Rockloff and Moore 2006), such that more suitable decisions in terms of ecological outputs are made at this level. Hence, this mechanism states:

Mechanism 1b: Participatory governance on higher scalar levels is conducive to environmental effectiveness.

When it comes to legitimacy, local decision-making is expected to be better able to generate representative and legitimate governance procedures (Dahl 1994; Loubier et al. 2005). This is due to the higher degree of commitment to, identification with, and interest in the local environment:

Mechanism 1c: The legitimacy of participatory decision-making is inversely related to the scalar level of governance.

Contrary to this mechanism, other scholars stress that the views and values of the general public, particularly interests that are not place-based (e.g. general welfare or ecological conservation), are likely to be better represented on larger geographic scales than at the local one, because with local scales, local self-interest tend to prevail, disregarding the lager common good (Soma and Vatn 2009). Furthermore, higher level processes were found to comply better with principles of representation and of professionalism overall, calling in to question the legitimacy of more local procedures (Rockloff and Moore 2006). Hence, this counter-mechanism states

Mechanism 1d: The legitimacy of participatory decision-making is positively related to the scalar level of governance.

#### Participation and Scalar Dimensions—Issues of Scalar Fit

Environmental problems typically are not confined by strict boundaries, and drivers for environmental processes may also be situated on different scales, all of which typically cut across political governance units. Such scalar “misfits” (Young 2002) between ecological and governance scales cause spatial spillovers and thus environmental ineffectiveness, which, given their cross-boundary nature, cannot easily resolved through a mere upscaling of governance-levels. The obvious response to such scalar tensions is that administrative scales should be adapted to ecological scales (Young 2002: 20, referring to Berkes and Folke 1998). Participation is regarded as a potential tool “to help us bridge the discontinuity between geographical and jurisdictional boundaries found in water resources management” (Delli Priscoli 2004), in particular if stakeholders manage to adapt flexibly to ecological scales (Cash et al. 2006; Delli Priscoli 2004). Such actors then play the role of intermediaries, operating between other actor groups given their ability to work as boundary organizations across different scales and contexts (Moss 2009):

Mechanism 2a: Participation of intermediary actors helps to bridge scalar misfits.

As regards legitimacy, the alignment of governance processes with hydrological boundaries is not unproblematic. Political institutions based on set territories with unequivocal membership draw on established mechanisms of legitimacy, primarily elections and representation. Functional jurisdictions such as watershed institutions, by contrast, lack a clear notion of membership and therefore tend to perform less well on classic criteria of representation (Meadowcroft 2002) and accountability (Huitema et al. 2009; Peters and Pierre 2005). This suggests that

Mechanism 2b: Participation on task-specific scales tends to suffer from problems of legitimacy as compared to participation on territorial scales.

#### Polycentric Governance Systems

In addition to the influence of individual scalar levels or dimensions, the overall configuration of the governance system has to be taken into account. Here, polycentricity refers to a system of many autonomous, independent, but interacting, decision-making bodies with overlapping jurisdictions (for an overview see: Aligica and Tarko 2012). This concept has widely been used to study natural resource governance (Andersson and Ostrom 2008; Ostrom 2010). Because they possess multiple decision points, it is argued that polycentric governance systems have greater flexibility than monocentric ones in the event of sudden changes, and their inherent redundancies are expected to produce a higher diversity of possible solutions (Folke et al. 2005; Ostrom et al. 1961), making these systems more effective:

Mechanism 3a: Polycentric systems are more effective, due to greater adaptability.

On the downside, polycentric systems are said to suffer from high fragmentation and co-ordination costs (Huitema et al. 2009). A polycentric—or, for that matter: fragmented—system may on the one hand be capable of solving its own, particular problems, but may be unable to address larger-scale challenges (Fung and Wright 2001). Hence, the counter-mechanism reads

Mechanism 3b: Polycentric systems are less effective, due to higher transaction costs.

When it comes to issues of legitimacy, multiple levels with different venues of decision-making also bear the risk of lacking transparency and problems of legitimacy (Benz 2001). These situations may lead to “responsibility floating” (Bixler 2014), where responsibility over environmental problems is constantly relocated by the actors within a polycentric governance system. Hence, we state that

Mechanism 3c: The number of decision-making levels and the overall complexity of the multi-level system have a negative effect on the transparency and legitimacy of the process.

## Implementing the WFD: Certainly Multi-Level, Somewhat Participatory, But Also Legitimate and Effective?

The WFD arguably constitutes the first principal EU policy that aims to achieve substantive goals *and* to enhance democratic legitimacy through deliberate re-scaling of governance (Moss 2004). Following the ‘mandated participatory planning’ approach (Newig and Koontz 2014), the WFD defines material goals (good water status for all EU member states’ inland ground and surface waters), which have to be met following elaborate procedural requirements. These entail the development of RBMP and Programmes of Measures (PoM) within a prescribed six-year planning cycle, assessing current water conditions and defining actions to be taken to reach the overall goal of good water status. These plans themselves serve as political programs stipulating and guiding river basin management and the implementation of measures in the respective river basin districts. Participation of non-state actors^1^ plays a vital role in this planning process. Relevant stakeholders and the public must be encouraged to input on the production and implementation of RBMP and PoM (with the first cycle plans due by 2009). In this, EU member states are given substantial leeway in how to operationalize and approach the overall goals and to design governance processes on this way (Newig et al. 2014).

### Case Selection and Research Methods

We investigate empirically the scalar particularities of WFD implementation, considering participation across different scales and levels. Our analysis is guided by the above-formulated mechanisms. We compare original empirical evidence from two case studies of participatory WFD implementation in the German states of North-Rhine Westphalia (NRW) and Lower Saxony (﻿LS), focusing on one sub-basin in each state. We selected these two states for a number of reasons. First, both are large states with a mix of urban and rural areas and a comparable population of more than 10 million inhabitants. Second, both have a different institutional legacy such that the WFD governance structure arguably will play out differently in both settings. Notably, NRW has administrative districts (Regierungsbezirke) as an intermediate level of government, which LS abolished in 2005; also, NRW has a long-standing tradition of powerful semi-public water associations, which LS and in fact most German states have not. Finally, data acquisition has been greatly facilitated by pre-existing relations of project members to a range of stakeholders in both case study regions.

As Table 1 illustrates, the two states organized WFD implementation in a complimentary way, with NRW concentrating participatory activities on local units below the sub-basin scale, while in LS the sub-basin scale served as the smallest governance unit. With its multiple levels of decision-making, this analytical set-up yields a substantial variety of more or less participatory decision-making processes on different scalar levels and with varying degrees of polycentric complexity.

**Table 1:** Participation mechanisms in the multi-level implementation system of the WFD, focusing on the two case regions. ‘Cons’ refers to the formal consultation processes mandated by Art. 14 WFD

	

We analyzed official documents such as RBMP, PoM, implementation timetables, and basin reports, including evaluations and assessments by EU and national agencies; further, secondary literature, internal memos, protocols, websites, and email correspondence. For a comprehensive understanding of the interests at stake, the participatory processes and their outcomes, we conducted guided interviews at the levels of the EU, Germany, the two federal states, and municipalities. A total of 27 interviews were conducted between April 2011 and February 2013 with decision makers and process organizers, as well as with representatives of interest groups and associations. They lasted on average between one and two hours. We performed a content analysis of the interview transcripts and the additional case material. Using the analysis software MAXqda, the case material was structured into context, process, output, and implementation (127 codes), and ordered by relevance for each mechanism. Quotes are marked in the text and can be found in the online supplementary material. In this way, we reconstructed the participatory processes and linked them to their respective substantial and social outputs.

### North-Rhine Westphalia

**Fig. 2:**
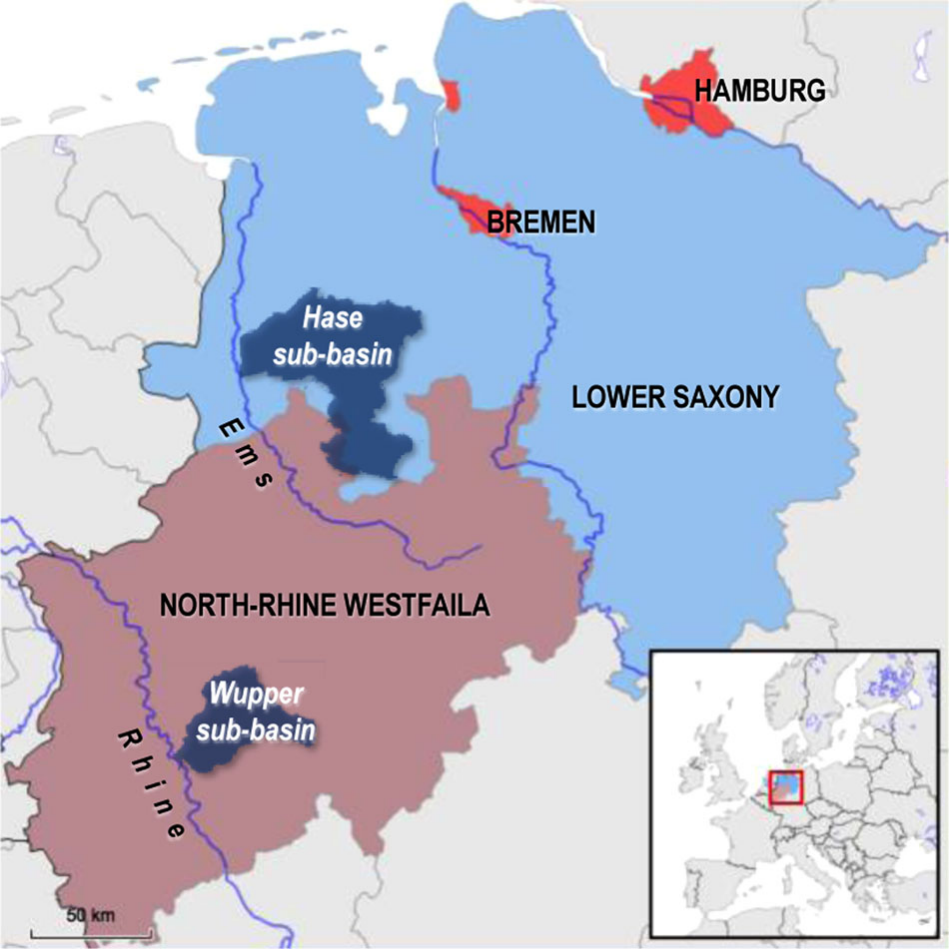
The case study regions of Lower Saxony with the Hase sub-basin and North-Rhine Westphalia with the Wupper sub-basin, located in the north-western part of Germany. Own drawing created with stepmap.de

In NRW, the state environmental ministry is charged with WFD implementation. In 2005, operative implementation was assigned to the four district governments. Our core example is the river Wupper, a tributary to the Rhine with a length of 115 km (see Fig. 2). The Wupper sub-basin is a heavily populated area with ~890,000 inhabitants. Diverse water uses (tourism, agriculture, industry, hydroelectricity) have led to some conflicts and environmental degradation in the past. The Wupper sub-basin cuts across the Düsseldorf and Cologne district governments, with a lead role assigned to the Düsseldorf government. For planning purposes, the sub-basin was further divided into three planning units of Upper Wupper, Lower Wupper, and Dhünn.

Aside from the district governments, a water board—the ‘Wupperverband’—is one of the principal actors in the area. This sub-basin-wide public body was established by the government in 1930 and is responsible for the main water management tasks (e.g., water body maintenance, wastewater treatment, and water supply). Municipalities, water utilities, and larger industrial enterprises are obligatory members of the Wupperverband and pay substantial fees (Moss 2012).

#### Round Tables

In NRW, stakeholders were involved in Round Tables between 2008 and 2009. In the Wupper area, these were organized by the district government of Düsseldorf and held at the level of the planning units Upper Wupper, Lower Wupper, and Dhünn. Their aim was to include local knowledge into the planning process (District Government Düsseldorf 2008a). Their output served as a proposal for the production of RBMP and PoM by the state environmental ministry.

The district government selected the participants, targeting mostly organized stakeholders related to water management. For example, the first Round Table of the planning unit Lower Wupper involved 21 participants, the second 49 participants, and the third 43 participants including representatives of district governments Düsseldorf and Cologne, the Wupperverband, municipalities, farmers association, an environmental NGO ('Na﻿turschutzbund Germany’ - NABU), land owners, infrastructure operators, water utilities etc. (District Government Düsseldorf 2008b). According to an interviewee, many participants primarily attended out of concern for negative consequences for their constituency (Interview LANUV, quote #1). One of the crucial points during discussions was the voluntary nature of the implementation process (Interview district government, quote #2).

It was agreed that only generic measures but no concrete proposals for action were to be included in the official PoM, while concrete measures were only documented in background papers (District Government Düsseldorf 2008a,c). The more detailed ideas for specific measures that were gathered during the Round Tables were transferred to the next stage, the ‘cooperations’, which are discussed below. One interviewee characterized the Round Tables as a place for information presentation by the organizers and general discussion, while little input was actually solicited from stakeholders (Interview district government, quote #3). Other interviewees felt that every participant was at least given the opportunity to voice his or her opinion or interest, and that these concerns were taken up for consideration (Interviews local agriculture association; water association, quote #4). Participants also reported that the Round Table allowed them to establish informal relationships with other stakeholders that made it easier to cooperate with them (Interview chamber of agriculture, quote #5) and to learn about the implementation process (Interview district government, quote #6).

#### Local Cooperations

In NRW, concrete planning, elaboration, and prioritization of measures to implement the WFD was done in local ‘cooperations’. In the Wupper area, three cooperations were created at the level of planning units, organized by the Wupperverband. In order to allow for intense participation, cooperations were sub-divided into a total of 10 working groups that covered small areas such as specific water bodies. Each working group had between 13 and 60 participants^2^. Following introductions into the planning process by experts of the Wupperverband or the water authorities, participants had the opportunity for in-depth discussion of measures using prepared maps and graphic tools (e.g., Wupperverband 2011). Between 2010 and 2012, among the three cooperations, a total of 25 workshops were conducted in which more than 100 stakeholders participated (Wupperverband 2012). Each cooperation summarized its planning results in a map containing detailed information on potential measures concerning their feasibility, priority, costs, and impacts. These results were incorporated into ‘implementation timetables’, planning documents that included the maps and lists of measures and which were published in 2012, serving as a concretization of the RBMP and PoM^3^.

#### Symposium

In 2010, the district government and the Wupperverband established a joint symposium for the whole Wupper area. This format resulted from a fusion of two separate but similar annual meetings, which previously had been held by each of the two public bodies on their own. The symposium is held annually and aims primarily at informing a broader audience of stakeholders on the current progress of WFD implementation, and secondarily at discussing issues of water resource management in more general terms.

#### Substantive Outcomes

For all German river basin districts, including those in NRW and LS, the official planning documents of RBMP and PoM turned out to provide only cursory information on concrete measures and their implementation (European Commission 2012). Hence, these documents do not lend themselves to substantially assess measures and action on the ground, let alone their attribution to the planning process and participation of stakeholders. In NRW, the environmental ministry compensated for sparse information in the planning documents by publishing more detailed ‘fact sheets’. These contain substantive outputs and potential programmatic measures in a more fine-grained way on the level of sub-basins and even catchments (NRW Ministry of the Environment 2009). Additionally, in the Wupper area, the implementation timetables contained concrete measures and actions on the ground (Wupperverband 2012). These identify more than 900 single measures for the Wupper’s three planning units covering diverse areas such as morphology, point source pollution, land use, or fishery. Measures span from efforts to enhance the information basis through further studies and monitoring to substantive and cost-intensive infrastructure measures, such as the relocation of riverbeds or the removal of artificial barriers. Despite the major expenditures that some of these measures entail, only nine measures were assessed as impossible, while almost 70 % had already implemented or deemed possible in 2012 by the Wupperverband, who is charged with implementation (Wupperverband 2012). With this, the Wupper ranges above the German average when it comes to measure implementation: a recent evaluation on behalf of the EU Commission (WRc plc 2015) for all German river basins estimated an average of 50–68 % being under implementation.

Water status in the Wupper sub-basin improved during the last WFD cycle between 2008 and 2014. The number of water bodies with good or better ecological status doubled in this time, constituting now around a quarter of water bodies in the sub-basin. At the same time the share of water bodies with poor or bad water status or potential decreased by 40 percent, indicating an overall positive trend. Main persisting issues in the sub-basin are hydro-morphological deficits, i.e., severely altered river beds offering only sparse aquatic habitats, passability for fish, and pressures from urban settlements (North Rhine Westphalia Ministry of Environment 2015). Attributing these positive developments in environmental quality directly to the processes and activities described above appears problematic, as alterations in river structures may show their environmental impact only after some time lag. Notwithstanding these caveats, improvements in water status often occurred regarding the river structure and habitats, which was targeted in multiple measures developed in the participatory processes.

### Lower Saxony

The responsible authority for WFD implementation is the LS Ministry of the Environment, with operative implementation carried out by the environmental state agency ('Niedersächsischer Landesbetrieb für Wasserwirtschaft, Küsten- und Naturschutz' - NLWKN). We focus on the Hase area, a sub-basin of the river Ems, which is characterized by agriculture and intensive livestock farming. Excess production of liquid manure has resulted in high pressure on ground and surface water due to high nitrate concentrations. Intensive agriculture has come to be a part of the region’s identity and “is reflected by the interests and perceptions of many actors involved in the implementation process of the WFD” (Kastens and Newig 2008).

#### Area Forums

The earliest major form of public involvement were the area forums, established by the environmental ministry. From 2004 to 2009, these were held annually in four hydrologically defined regions, with up to 100 participants attending each meeting. The ministry used the area forums to give an account of the overall progress of the implementation process and of technical aspects. These formats were criticized as lacking sufficient feedback possibilities on the part of participants (Ridder et al. 2007).

#### Area Cooperations

As a more active form of involvement, LS established 30 local ‘area cooperations’ in 2005, on the level of sub-basins. According to a ministerial decree (Lower Saxony Ministry of Environment 2005), area cooperations were designed to accompany the whole WFD implementation process. However, no formal decision-making competence was transferred to the participants, which was seen critically in an earlier assessment (Kastens and Newig 2008). The state government provided each cooperation with an annual budget of 15,000 Euro for implementing measures (Kommunale Umwelt-AktioN U.A.N. 2008). The area cooperation covering the Hase sub-basin met several times per year from 2006 to 2009 and just annually thereafter. Initially, it was intended to involve one representative of each stakeholders group, such as administrative counties and municipalities, farmers associations, business, water boards, environmental NGOs, and regionally specific actors (e.g., dyke associations, fisheries). However, as municipalities could not agree on one representative, several were accepted. Other organized interests such as the fisheries requested inclusion in the Hase area cooperation. Some interviewees saw this as a clear disadvantage because larger groups made discussions more difficult and harder to moderate (Interview maintenance association, quote #7), reducing the possibilities for dialogue and discussion (Interview environmental organization, quote #8). In contrast to the municipalities, environmental organizations had problems finding a capable representative for each area cooperation (Interview environmental organization, quote #9).

During the Hase area cooperation meetings, different interests of participants became apparent: Agricultural representatives and water maintenance boards stressed the function of flowing water bodies for agriculture, seeing little room for space intensive and costly natural development of water bodies (Interviews maintenance organization; agricultural association, quote #10). Some of the municipalities and other stakeholders saw the WFD as a chance to stress the rivers’ use for people’s well-being, recreational interests and tourism (Interview county; environmental organization, quote #11, see also quote #20). The tension between (agricultural) land use and environmental protection created some controversy, but no heated conflicts were reported (Interview water treatment, quote #12). A great obstacle to the whole process was the unresolved question of financing because only few small measures could be implemented with 15,000 Euro per year (Interview maintenance association; agricultural association, quote #13). In terms of capacity building, three maintenance associations in the sub-basin regions formed an umbrella organization in order to increase the capacity to implement measures and the municipalities established means of information exchange. The interviews indicate that participants valued the input of information, provided mainly by the NLWKN, as well as the opportunity to get to know the interests and positions of other stakeholders (Interview maintenance association; water treatment, quote #14).

#### Substantive Outcomes

Although participants contributed to the compilation of lists that named concrete measures (Interview NLWKN, quote #15), these were not included in the final RBMP or PoM, which listed generic measures only, similar to those in NRW. This is in line with findings from other area cooperations (Koontz and Newig 2014) and an EU evaluation of all German basins (European Commission 2012). The NLWKN published data sheets for each water body, listing core pressures and prioritized measure suggestions fitted to these^4^. For the Hase, these sheets suggested around 300 measures, covering areas such as morphology, connectivity, point source, and especially also diffuse source pollution. Furthermore, the area cooperation assisted in the declaration of water bodies as natural, artificial, or heavily modified. After some discussions, a large number of water bodies were classified as heavily modified (HMWB) (Interview environmental organization, quote #16).

Actual implementation of measures was and is based on a voluntary model. Action on the ground depends on the commitment and engagement of local governments, authorities, and stakeholders in the basin, who are encouraged to implement identified measures with financial support from the state government (Koontz and Newig 2014). However, potential co-implementers felt that this WFD process had complicated the implementation of measures due to an increase in bureaucracy (Interview maintenance association, quote #17). Furthermore, this decentralized procedure resulted in the disregard of major issues of agricultural pollution, notably nutrients. A recent EU-ordered evaluation of WFD implementation in Germany (WRc plc 2015) found for the Ems basin as a whole that for more than 85 % of all measures to reduce nutrient pollution in agriculture—beyond the requirements of the Nitrates Directive—implementation has not yet started until 2012. On the other hand, implementers reported some progress in the revitalization of river banks (Interview fishery, quote #18) and the removal of other obstacles (Interview maintenance association, quote #19). Many municipalities saw the WFD as an opportunity to conduct projects that combined the aim of natural development with other objectives such as creating value for tourism or ensuring flood protection (Interview municipal association, municipality, quote #20).

The actual impacts of these measures for the water status in the sub-basin and beyond are hard to assess. Surface and groundwater assessments undertaken in 2014 as part of the subsequent WFD planning cycle show that only 1 % of all surface waters of the whole Ems basin is of good or better ecological status with more than 80 % of poor or bad ecological status or potential. In the Hase sub-basin, only 2 out of more than 70 water bodies acquired good ecological status. Compared to the 2008 assessment, improvements are marginal. Groundwater quality even deteriorated in one of the aquifers in the sub-basin. Main pressures continue to be diffuse pollution from agriculture as well as river development and construction (Lower Saxony Ministry of Environment 2015, NLWKN 2009, 2012). These not only affect the water status in the basin but also contribute to considerable eutrophication in the German and Dutch North Sea coastal waters (Bund-Länder Arbeitsprogramm Meeresumwelt 2011).

## Discussion in the Light of the Mechanisms

Having examined the different participation mechanisms employed in the two case regions in some detail, we now take a more analytical perspective and relate these findings to the mechanisms formulated at the outset (Table 2).

**Table 2:** Summary of mechanisms and related case study evidence

No.	Mechanism	North Rhine-Westphalia/Wupper	Lower Saxony/Hase
M1a (b)	The *smaller (higher)* the scalar level of participatory governance, the greater the environmental effectiveness of decisions.	In support of M1a: Cooperations on catchment level allowed stakeholder knowledge to bring in knowledge through detailed maps and timetables, tailoring measures around local conditions.	In support of M1a: Area cooperations on sub-basin level gave stakeholders the opportunity to contribute to lists of measures, adding to the consideration of local conditions.
		–	In support of M1b: Environmental groups in particular had limited capacity to participate meaningfully and voice ecological concerns in local processes.
M1c (d)	The *smaller (higher)* the scalar level of governance, the greater the legitimacy of decisions.	In support of M1d: Lay stakeholders faced capacity problems to ensure representation in all local venues.	In support of M1d: In area cooperations, lay stakeholders faced capacity problems to ensure representation in all venues. Inclusion of all relevant local governments on the sub-basin scale led to hampered discussion quality.
		In support of M1c: In processes on catchment scale, participants could voice their concerns and effectively relate to water management issues.	In support of M1c: Local fieldtrips increased participants ownership of processes.
M2a	Participation of intermediary actors is likely to bridge the *misfit* between ecological and administrative scalar dimensions.	In support of M2a: The Wupperverband as historically grown institution embedded in established administrative structures served as important intermediary bridging misfits.	In support of M2a: The inclusion of stakeholders organized on hydrological scales (e.g., maintenance associations) helped to communicate between the different scalar dimensions.
M2b	Participation on *task-specific* (i.e., hydrological) scales tends to suffer from problems of legitimacy as compared to participation on territorial scales.	Against M2b: Relying on a multi-layered structure on sub-basin and catchment levels, a compromise between inclusiveness of the process and a manageable process design could be found.	In support of M2b: Given the intersection of the Hase sub-basin with multiple municipalities, there appeared a trade-off between full representation of every stakeholder and a proper group size and working climate.
M3a (b)	Polycentric systems are *more (less)* effective.	Mixed evidence: Planning competences are dispersed between district governments and water boards, further delegated to various participatory venues (planning polycentricity).	Mixed evidence: Polycentricity could mainly be found for the implementation phase, which was decentrally organized.
		In support of M3a: This allowed for the inclusion of various interests and sources of knowledge and the identification of a large number of specific measures.	In support of M3a: Implementers (e.g., municipalities, maintenance boards) could realize some locally tailored measures.
		In support of M3b: Implementation was guided more strongly by central actors.	In support of M3b: Increased transaction and coordination costs could be observed.
M3c	The *number of decision-making levels* and the overall complexity of the multi-level system have a negative effect on the transparency and legitimacy of the process.	In support for M3c: Multiple venues hampered appropriate representation of every actor.	In support for M3c: Decentralized implementation system proved in transparent.

### Participation at Small vs. Large Scalar Levels

Intensive, interactive forms of participation were mostly organized on a sub-basin level or, in the case of the Wupper, in cooperations on an even more local level. Less intensive forms of participation such as formal consultations, the Council, and in LS the area forums were organized on higher spatial levels. Interviews suggest that participants often found the group size too large for meaningful discussion, but overall had the possibility to voice their interests (Interview maintenance association, quote #8). Intensive deliberations mostly took place in very local settings, such as working groups.

Regarding *effectiveness* (M1a and M1b), our findings provide some evidence that *local* participation is conducive to WFD planning processes. In the Hase case, key stakeholders’ knowledge on different aspects of water management proved valuable for naming measures and compiling lists, as well as for capacity building (all of which would not likely have been possible on a more aggregated governance level). Even more so, the cooperations in NRW, which were held on yet more local scalar levels, succeeded to include local knowledge by providing detailed maps and implementation timetables and working on specific water bodies. The NRW approach thus re-scaled participation from the sub-basin down to a more local level in order to enhance effectiveness, particularly regarding process quality and outputs, thus, supporting mechanism M1a. In support of M1b, however, it became apparent that the LS area cooperations were situated on too local a level for environmental groups to meaningfully engage. The fisheries representative in the Hase area, for example, did not represent the full spectrum of environmental concerns and was, notably, no expert in nutrient issues. On a more aggregated spatial level, such as the state, environmental groups are organized more professionally, not having to rely on voluntary engagement. Whether or not M1a or M1b holds thus appears to depend on whether participation is to mainly solicit environmental knowledge (favoring local processes—M1a) or whether it seeks to promote environmental advocacy (favoring less local processes—M1b). The dilemma of the LS approach, then, was that the area cooperations tried to achieve both which they could not. NRW, however, with its more flexible and multi-leveled approach of both soliciting local knowledge *and* allowing for effective NGO representation at more aggregated levels, proved superior in effectiveness terms.

As regards the *legitimacy* of decision-making, the analysis indicates that the re-scaling to rather local decision-making does benefit participants’ commitment and identification with the area and the possibility to address specific issues during the participation process (M1c). However, in the Hase region, stakeholders expressed that the area cooperation was still covering too large an area to identify with the whole region and to discuss particular measures (Interview environmental organization, quote #21). The larger group size resulting from this wider geographic scope further hampered the discussion climate. To strengthen identification among the participants, the area cooperation organized field trips to particular water bodies, which stakeholders were chiefly interested in and which corresponded to their more local sense of place (M1c). In the Wupper case, the cooperations established working groups on an even more local scale, where affected stakeholders had access to and could discuss measures for specific water bodies. This increased ownership on behalf of the participants and led to a high appreciation of the process (Interview nature protection, quote #22).

At the same time, these small-scale participatory processes revealed deficits concerning input legitimacy (representation) (providing support for M1d): In both Wupper and Hase cases, environmental organizations relying on voluntary action by their members (as is typical for local environmental organizations) faced difficulties to represent their interest in each venue. In the Hase region, environmentalists were not able to nominate a member of a genuine environmental group. In the Wupper case, stakeholders from voluntary organizations also reported difficulties to attend activities they were invited to (Interview local agricultural association, quote #23). These findings suggest that highly local participation overburdens voluntary organizations regarding their personnel, time and financial resources—a phenomenon which would likely have been less pronounced in participatory formats on more aggregated levels.

To conclude, we find mixed results regarding Dahl’s (1994) proclaimed dilemma between legitimacy through participation (more likely to be attained in local decision-making) and effectiveness (more likely to be attained at more aggregated levels). In fact, both effectiveness and legitimacy were scale-dependent but not in a straightforward way. Contrary to Dahl’s expectation, local processes did appear effective in the sense of information-gauging and working toward implementation of measures (as opposed to a counterfactual situation of tackling these issues at a more aggregated scale). However, the actual WFD planning documents, that were to address the overall water-related problems in a larger unit, lacked the rigor and concreteness to be effective, e.g., in terms of tackling the overall nitrate problem in LS. Thus, we find a trade-off between vague outputs (plans) and more effective outcomes (implementation). In terms of legitimacy we found that the more local, the more stakeholders identified with and accepted decision-making processes but that very local processes did less well regarding access and representation of groups (in particular NGOs). The key scale-related trade-off thus appears not one between legitimacy and effectiveness, but between the different dimensions within the broader concepts of both effectiveness and legitimacy, thus challenging conventional assumptions.

### Scalar Fit

Our findings suggest that in both cases a misfit between hydrologically and politically delimited institutions was of concern. Both the Hase and the Wupper sub-basins cross the jurisdictions of multiple local authorities, creating misfits between political scales of interest representation and the newly introduced, hydrologically oriented governance units. According to mechanism M2a, such misfits are likely to be bridged through participation. In the Hase case, some stakeholders such as the maintenance associations, the environmental NGO and the water utilities are in fact organized along hydrological boundaries. As intermediaries, their participation partly helped to communicate between the logic of sub-basin management and the logic of municipal and county administration. In the Wupper case, the misfit between political and hydrological scales is less pronounced because the Wupperverband has a long-grown structure accommodating the hydrological scale dimension. This association, therefore, served as important intermediary between the political institutions and the different processes on the water body level, such as the local cooperations.

With regard to M2b, functionally delimited institutions such as those on hydrological scales are suspected to suffer from a lack of legitimacy. Our case studies partly support this mechanism. In LS, the area cooperations, while cutting across established territorial boundaries, in theory allowed only one representative of each jurisdictional level (Lower Saxony Ministry of Environment 2005). Consequently, counties and municipalities did not feel appropriately represented in this setting (Kommunale Umwelt-AktioN U.A.N. 2006a, b; Lower Saxony Ministry of Environment 2006). This eventually led to an expanded group, allowing for extended representation at the expense of a less productive working atmosphere. In the Wupper basin, a successful attempt was made to circumvent the M2b problem through a multi-layered structure that allowed for greater stakeholder inclusion (see also Hüesker and Moss 2015). Representation was further enhanced by the targeted selection of stakeholders for different arenas following a stakeholder analysis (Seecon 2007).

Our findings thus suggest that scale-adapted governance on functionally delimited scales pose a challenge to legitimacy. We may reasonably assume that on those local scales relevant to our study (sub-basin), pollution spillovers (e.g., regarding nitrate pollution of ground and surface waters) are not so pronounced that governance on hydrological scales will actually outperform. Where stakeholders are organized according to ecological boundaries, problems of legitimacy will result.

### Polycentric Governance System

**Fig. 3:**
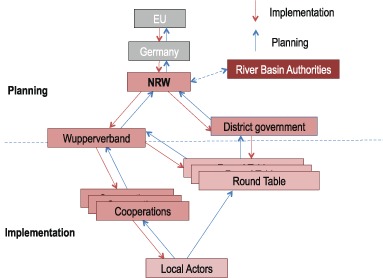
WFD implementation structure in North-Rhine Westphalia

**Fig. 4:**
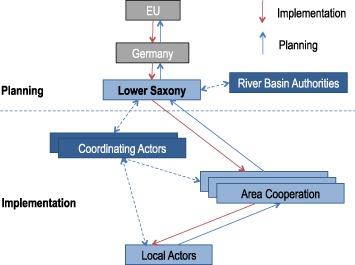
WFD implementation structure in Lower Saxony

With regard to the overall governance system put into place for WFD implementation and its polycentricity, we find substantial differences between LS and NRW. To understand the relevant differences, it is useful to distinguish the institutional set-up of the *planning* process (i.e., the preparation of RBMP and PoMs) and that of the process of *implementing* measures on the ground (illustrated in Figs. 3 and 4).

In LS, planning has been essentially centralized and mainly in the hands of the environmental ministry and the NLWKN, whereas the influence of participatory area cooperations remained limited. Implementation of measures, on the other hand, has been organized decentrally. Many different actors, such as water maintenance boards and municipalities are expected to assume responsibility for the implementation of measures, using the area cooperation as a means of information exchange (Kommunale Umwelt-AktioN U.A.N. 2006a). Interviews reveal that some of these actors indeed realized measures, reacting flexibly to local circumstances (in support of M3a). On the downside, and in support of M3b, interviewees point to the coordination costs of such a decentralized and fragmented system, such as the municipalities who initiated a coordinating body (Interview county, quote #24).

In NRW, the WFD implementation system is both more and less polycentric than in LS: while planning is more polycentric, implementation is less so (cf. Fig. 3). Different from LS, WFD-related *planning* competencies are distributed over the district governments as well as the water boards such as the Wupperverband (in those sub-basins in which they exist). Both structures compete but also collaborate, this being a typical indicator of polycentricity. The numerous participation mechanisms put into place on different levels and which contributed to the planning efforts, added to this polycentricity. The overall effect of this polycentricity on planning quality is certainly difficult to measure. However, the elaboration of implementation timetables, which identify very concrete measures (as opposed to planning in LS), is clearly a comparative ‘success’, and arguably due in part to the integration of various sources of knowledge by local stakeholders. Moreover, the Wupperverband, competing with the district government, assumed a leadership role, provided considerable resources and served as a source of innovation (Hüesker and Moss 2015). *Implementation* of measures, again different from the LS model, was guided more strongly by central actors (Wupperverband, district government) rather than leaving it solely to local actors. Considering the overall positive trend in the ecological water statues, there is no indication to believe that this model was less ‘successful’ as compared to the LS approach in terms of progress toward WFD goals.

Are polycentric systems less legitimate in terms of representation and accountability (M3c)? On the one hand, the complex participatory structures did increase input-oriented legitimacy as compared with the pre-WFD situation in both case regions. In NRW, where polycentricity was highest in the WFD planning realm, competing structures (Wupperverband vs. district government) may have decreased transparency and thus accountability. However, the Wupperverband was legitimized by formal decisions by its members. Legitimacy suffered somewhat because the multitude of different venues for participation was overwhelming for some of the actors, making it difficult for them to decide where to participate. In LS, the high polycentricity in the implementation realm, closely linked to a lack of central co-ordination or guidance of implementation, entailed a lack of transparency of what measures are implemented by whom as well as a potential withdrawal of state responsibility.

## Conclusions

The European WFD attempts to rescale competences of water governance in a newly fashioned multi-level system of mandated participatory planning. This constitutes an experiment for governments, involved stakeholders and citizens across the European Union. German federalism has produced 16 such experiments, as each federal state pursues its own strategy of setting up participation mechanisms. Our comparative study of two such cases reveals, first, that the WFD did impact on institutionalizing hydrological governance scales and participation. Participation has been put in place in various forms and on multiple levels of governance, showing distinct differences between the two studied cases. Contrary to expectations, governance competences have scarcely shifted toward hydrological scales but remain with the federal states, with limited cross-border cooperation in river basins. In NRW, the Wupperverband, acting on sub-basin scale, has been strengthened and partly assumed competences originally held by the district governments. In LS, area cooperations were implemented on sub-basin and catchment scales, but had little influence on planning.

Did the WFD succeed in improving both effective water governance and democratic legitimacy of decision-making through its re-scaling approach? Or do scale-related dilemmas prevail?

As regards Dahl’s (1994) ‘democratic dilemma’ and the local—supra-local dualism, the message taken from the case study comparison is not unequivocal: Given the complexity of water management issues to be tackled for WFD implementation, the more local decision processes appeared both more effective (in the sense of producing better informed and more meaningful outputs) and more legitimate (in terms of relating closer to citizen and stakeholder interest). On the other hand, local processes in LS were more susceptible to being dominated by economic (agricultural) interests, working against strict water protection. To a lesser degree, the argument of greater effectiveness of higher-level decision-making proved relevant, namely the positive effect of central guidance on measures implementation in NRW (which was largely lacking in LS). This relative superiority of local as opposed to higher-level decision-making must, of course, be interpreted against the more local nature of most water management issues encountered in the two case regions. A key factor determining whether local or less local processes are more effective depended on whether environmental knowledge or environmental advocacy is sought.

Was participation able to bridge ‘misfits’ between ecological (i.e., hydrological) and administrative scales of governance, or did this introduce new problems of legitimacy? Water-related, task-specific governance scales conflicted with established notions of territorially based representation and legitimacy, thus creating scalar ‘misfits’. This was more pronounced in LS, with area cooperations crossing territorial boundaries, as compared to the NRW-model, in the Wupper case due to the strong role of the grown basin-oriented water board. While the participation of actors organized along the sub-basin boundaries in the Hase did appear to bridge misfits, problems of legitimacy and representation remained. Whether participation helps to bridge scale-related misfits appears to depend on the institutional history, with grown structures more likely to perform than fresh reforms of re-scaling.

Polycentricity, finally, appeared somewhat favorable in effectiveness terms. Our analysis suggests to distinguish between governance polycentricity of the planning system and that of the implementation system. Higher polycentricity in planning in NRW proved successful due to competing structures, while higher polycentricity in implementation in LS proved less conducive to both effectiveness and legitimacy. Clearly, this distinction between the planning and the implementation stage will warrant further enquiry.

Three caveats must be mentioned with regard to our assessment. First, contrary to earlier expectations, it has become apparent that the official planning documents (RBMP and PoM) were not used as the central vehicle for the development and implementation of measures on the ground, but rather as a means to symbolically report to the Commission. Instead, the initiated governance mechanisms triggered activities such as the elaboration of additional plans (implementation timetables in NRW) or the promotion of voluntary initiatives (LS) (see Koontz and Newig 2014). Second, the environmental impact of the studied processes and their outputs cannot yet be fully evaluated. Ecological data is still sparse and often real impacts of implemented measures become visible only after some time. Finally, the relevance of the hydrological scales involved in this case study (and similar others) is arguably questionable (Ingram 2011) and ultimately remains an empirical issue. This points to the politics involved in the re-scaling of governance, which has been highlighted in the critical human geography literature (Hüesker and Moss 2015).

Beyond, but related to the initial assumptions contained in the three sets of mechanisms, three key insights emerge from this empirical study. First, a major re-scaling effort such as the one introduced by the WFD cannot easily resolve scale-related trade-offs between effectiveness and legitimacy. Rather, grown, co-evolved institutional structures appear more important than ‘optimized’ scalar governance arrangements. Second, the dualism of effectiveness vs. legitimacy appears less pronounced than potential trade-offs between dimensions within either concept. Third, the concept of polycentricity appears more diverse than initially assumed and can be disentangled into polycentricity in planning and polycentricity in implementation.

The findings reported here are of wider importance to related attempts at governance re-scaling through mandated participatory planning. Such new governance modes appear, for example, in the Floods Directive, that mandates flood risk management plans to be produced until 2015 on the level of flood-risk areas (Newig et al. 2014), regarding the biodiversity regime (Paavola et al. 2009), or the Ambient Air Quality Directive (Newig and Koontz 2014). Research on scalar, multi-level, and participatory governance will, therefore, continue to be relevant beyond the implementation of the WFD.

## Electronic supplementary material

Supplementary Information

## Footnotes

1 Non-state actors are understood as all stakeholders from civil society, including citizens, and from private business, including farming, thus excluding actors from government and administration.

2 See https://www.wupperverband.de/internet/mediendb.nsf/gfx/6B5A7EA631FBD243C12579E400417AC8/$file/Text%20Begrue%C3%9Fung.pdf. (last accessed 14 July 2016).

3
http://wupperverband.de/internet/web.nsf/id/pa_de_planungseinheiten.html (last accessed 14 July 2016).

4
http://www.nlwkn.niedersachsen.de/wasserwirtschaft/egwasserrahmenrichtlinie/flussgebietseinheit_ems/hase/wasserkoerperdatenblatt/wasserkoerperdatenblaetter-fuer-die-gewaesser-im-bearbeitungsgebiet-hase-112997.html (last accessed 21 September, 2015).
