# Supplementary material for: Disentangling Puzzles of Spatial Scales and Participation in Environmental Governance—The Case of Governance Re-scaling Through the European Water Framework Directive
Source: Environ Manage. 2016 Sep 20;58(6):998–1014. doi: 10.1007/s00267-016-0753-8 (PMC5085982; doi:10.1007/s00267-016-0753-8)
Supplement: Supplementary file 1 — Supplementary Information [file 267_2016_753_MOESM1_ESM.docx]

*Online supplementary material to*

**Disentangling puzzles of spatial scales and participation in environmental governance – The case of governance re-scaling through the European Water Framework Directive**

*Authors:*

Jens Newig

[newig@uni.leuphana.de](mailto:newig@uni.leuphana.de)

Daniel Schulz

[Daniel.schulz@uni.leuphana.de](mailto:Daniel.schulz@uni.leuphana.de)

Nicolas W. Jager

[jager@uni.leuphana.de](mailto:jager@uni.leuphana.de)

*Affiliation of all authors:*

Research Group on Governance, Participation and Sustainability

Leuphana University of Lüneburg

Germany

*Contact address:*

Scharnhorststrasse 1

21335 Lüneburg

Germany

Ph: 0049-4131-6771726

Corresponding author: Nicolas W. Jager

**Table 1: Original interview quotes in German and their English translation**.

| No | German original | English translation |
| --- | --- | --- |
| #1 | “Es waren halt Akteure da, mit denen man vorher nichts zu tun hatte. Ich hatte aber den Eindruck, die haben nicht teilgenommen, um ihre Machtposition da irgendwie auszubauen, sondern um zu gucken: Droht mir irgendetwas? Wollen die mir etwas wegnehmen? Wollen sie meinen Gewinn mindern, oder wollen sie mir wie in der Landwirtschaft neue Regeln aufdrücken, neue Probleme schaffen? Also nicht in dem Sinne, dass man da dachte, man bringt sich ein und kann seine Machtposition… hatte ich eigentlich nie den Eindruck. Nur so: Inwieweit bin ich betroffen, muss da gegensteuern?“ (LANUV, S.12) | There were stakeholders present with whom we weren’t in contact before. However, I had the impression that they weren’t there to strengthen their position of power but rather to see: Is there something threatening for me? Do they want to take something away from me? Do they want to lower my profit or do they want to impose new rules, cause new problems, like in agriculture? Not in the sense that one thought, one can participate and [can strengthen] one´s position of power…I never got this impression. It was rather like: In what way am I affected, do I have to take action? |
| #2 | “Es ist ja so, dass der Bewirtschaftungsplan und das Maßnahmenprogramm behördenverbindlich sind. Das heißt also behördenverbindlich für die Bezirksregierung, für die unteren Wasserbehörden, aber auch für die Kommunen. Dass ein Zwang dahinter steht, und zwar der Zwang, die Ziele der Wasserrahmenrichtlinie spätestens 2027 unter Ausschöpfung aller Verlängerungsmöglichkeiten zu erreichen. Auf der anderen Seite ist es immer so von Landesseite propagiert worden, dass der Umsetzungsprozess ein freiwilliger Prozess ist, dass die bösen Worte wie Enteignung und so nie in diesen Prozess nie in diesen Prozess eingebracht werden. Und Sie merken ja schon, da gibt es ein Spannungsfeld. Denn letztendlich, wenn man es zu 100% freiwillig umsetzen würde, [...] würden die Ziele niemals erreichbar sein, das muss man ganz klar sagen. Das heißt, angestrebt wird so viel Freiwilligkeit wie immer möglich ist, aber die Grenzen dieser Freiwilligkeit, die werden durch den Ablauf des Umsetzungsprozesses schon gesetzt. Mit anderen Worten, wenn man erkennt, da ist jemand, der bewegt sich überhaupt nicht, der muss sich aber bewegen, damit die Ziele erreicht werden, dann werden schon irgendwo die Schrauben bisschen angezogen. Da hat es eine Reihe von Leuten gegeben, die sich erst dann bewegt haben, nachdem dann der Druck doch ein bisschen größer geworden ist. Die vertreten möglicherweise, ob sie es mittlerweile auf Überzeugung tun, oder ob sie es tun, weil sie es tun müssen, die vertreten sicherlich ähnliche Positionen wie diejenigen, die sich freiwillig so gewandelt haben, aber ist schon unterschiedlich, wie sie da hingekommen sind.” (district government, #80:59#) | The thing is, the river basin management plan and the programme of measures are binding for public authorities. Which means they are binding for the district government, for the lower water authorities but also for the municipalities. Which means that there is pressure behind all that, the pressure to achieve the goals of the Water Framework Directive at the latest by 2027, using all the given opportunities for extension. On the other hand, the [federal] state government has always put forward that the implementation process is voluntary, that evil words such as expropriation were never used in this context. And you will notice, there is an area of conflict. Because ultimately, if you want to implement this on a 100% voluntary basis, you would never achieve the goals, this is very clear. That means, it is aspired to have as much voluntariness as possible but there are limits to this voluntary nature which are set in the course of the implementation process. In other words, if one notices that there is somebody who is not moving but who should act in order to achieve the goals, there are some ways to exert pressure. There were a couple of people who only acted after the pressure got more intense. They probably have a similar position as the people who voluntarily changed, either out of conviction or because they have to, but there was a different way of getting to this point of view. |
| #3 | “Also es ist so, dass die Tagesordnung der Runden Tische, und die bestimmt ja natürlich letztlich auch den Inhalt, dass [die] Tagesordnung schon von den Organisatoren vorgegeben wurde. Das hängt damit zusammen, dass die Runden Tische nur zum Teil Runde Tische zum Diskutieren [waren]. Und zum großen Teil, würde ich sogar sagen, eine Plattform zum Informieren waren. Weil dieser Prozess der Umsetzung der Wasserrahmenrichtlinie war ja ein Prozess, der sich vom Ministerium über die Bezirksregierungen zu den Kommunen, zu den Wasserverbänden erstreckt hat. Und da die Bezirksregierungen in der Mitte dieser Kette sitzen, ist es eigentlich immer so gelaufen, dass wir die Runden Tische benutzt haben, um die Informationen, die vom Umweltministerium kamen, die nächsten Schritte, wie geht es weiter, dass wir die da weitergegeben haben. Umgekehrt haben wir natürlich auch, muss man sagen, die Stimmung auf den Runden Tischen aufgefangen und in Form von Dienstbesprechungen an das Umweltministerium herangetragen haben.” (district government, #84:55#) | The thing is, the agenda of the round tables, which ultimately determines the content, this agenda is set by the organiser. This has to do with the fact, that the round tables were only partly for discussion. And in large part, I would say, they are more a platform to inform people. This is because the implementation process of the Water Framework Directive was a process that extended from the ministry to the district governments all the way to the municipalities and water boards. And because the district council is located in the centre of this chain, we usually used the round tables in order to give people the information that we got from the Ministry of Environment and inform them of the next steps that had to be taken and the way we would proceed. On the other hand, we used the feedback of the round tables and reported this back to the ministry of environment during our meetings. |
| #4 | “Ich sag mal die Naturschutzverbände waren anfangs etwas engagiert oder engagierter. Aber das hat sich alles eingependelt. Es ist keiner vergessen worden, alle konnten ihre Meinung vertreten, es wurde fast auf alles Rücksicht genommen, was so machbar war.” (local agricultural association, #21:42#)  “In diesen Runden ist sehr produktiv diskutiert wurden, eigentlich von allen Beteiligten. Das war auffallend, dass nicht jeder unbedingt seine Fahne verteidigt hat, sondern auch Verständnis für die anderen Belange hatte.” (Water association, #15:40#)  “Wo es um unsere Themen ging, wo wir beteiligt waren, da waren schon die Akteure, die man sich vorstellen konnte und da auch beteiligt waren, die haben sich auch... ich denke auf Augenhöhe ist das diskutiert worden. Und sie haben sich auch da mit eingebracht und ich glaube auch, dass da keiner jetzt als der "Verlierer" rausgegangen ist. Ist mir in meinem Umfeld nicht bewusst geworden.” (Water association, [#21:39#)](http://localhost:2300/file=C:/Users/Ramona/Documents/Arbeit%20-%20Interviews/interviews/STE-006%20(2).mp3time=1299800) | I would say that the nature conservation organisations were more engaged at the beginning. But this evened out over time. Nobody was forgotten, all could state their opinion, almost everything that was possible was taking into consideration.  During these rounds, every participant was contributing to the discussion in a productive way. It was striking that not everybody was merely depending their point of view but the participants were also very understanding for other concerns.  When it came to topics in which we were involved, there were these stakeholders present that you would imagine and they were also participating. … I think the discussion was at eye level. And they did contribute and I don’t believe that anyone left as a “loser”. At least not in my environment that I was aware of. |
| #5 | “Q: Wie haben sich dadurch so die Akteurskonstellationen verändert zwischen allen Akteuren, die wir jetzt so durchgegangen sind?  A: Da muss ich sogar etwas Positives sagen. Denn Landkreistag, Städte- und Gemeindebund und wie die alle heißen, die sieht man da. Aber auch andere, zum Beispiel wieder eine IHK. Und da sagt man: Mann, ich hatte doch folgendes Problem. Wenn ich Sie gerade sehe, kann ich Sie da noch einmal eben drauf ansprechen? Oder haben Sie eine schnelle Antwort, oder darf ich Ihnen eine Email schreiben? Heißt also, unsere Gespräche sind nicht nur konzentriert auf Umsetzung Wasserrahmenrichtlinie, wir haben ja nebenbei noch etwas anderes zu tun. Und da ist durch diese Runden Tische und landesweiten Arbeitsgruppen und wie die Dinger alle heißen, ist man sich vertrauter geworden zwischen den Institutionen. So lästig dieses manchmal ist, und wie viel Arbeitszeit dieses bindet, aber diese Querverbindung, die ist dadurch deutlich besser geworden. “ (Chamber of agriculture, #31:47#) | Q: How did the constellation between stakeholders change, regarding all the stakeholder that we mentioned so far?  A: I must say something positive about that. Because you do see the association of counties, the association of towns and municipalities and whatever you might call them, you see them all there. But you also see others, for example chambers of industry and commerce. And you say: I did have this problem, now that I see you here, could we go over it quickly? Or do you have a quick answer or can I maybe send you an email? Meaning, our talks are not only focused on the implementation of the Water Framework Directive, we do have other things to do as well. And through these round tables and [federal] state-wide working groups and all those things, the institutions became more familiar with each other. However inconvenient it sometimes might be and the amount of work that goes into it, the interconnection actually clearly improved through it. |
| #6 | “Was man generell anmerken muss, ist, dass auf den Runden Tischen im Grunde genommen die Bereitschaft bei den wasserwirtschaftlichen Akteuren erst einmal hergestellt worden ist, um in so einen Prozess, Umsetzungsplanung mit konkreten Maßnahmen einzusteigen. Ich glaube, dass wir die Umsetzungsfahrpläne und die Organisation der Umsetzungsfahrpläne, dass wir die nicht so gut hingekriegt hätten, wenn wir die Runden Tische nicht gemacht hätten. Denn die Leute sind auf den Runden Tischen sensibilisiert worden für den gesamten Umsetzungsprozess. Und als es dann in die Verfeinerung ging, in die Konkretisierung des Umsetzungsprozesses, da waren die soweit sensibilisiert, dass sie in der Lage waren zu sagen: Ja, jetzt kann ich da auch, in welcher Form auch immer, mitarbeiten. Insofern haben die Runden Tische schon eine ganz wichtige Schlüsselfunktion im Umsetzungsprozess erfüllt, das muss man schon sagen.” (district government, #91:05#) | What one can generally note is that the round tables essentially established the willingness of those stakeholders concerned with water management to enter such a process of implementation planning with concrete measures. I think, we wouldn’t have been able to manage the implementation timetables and the organisation of the implementation timetables as well without the round tables. Because the round tables sensitised the people for the whole implementation process. And once we got to the refinement, to the concretisation of the implementation process, people were already sensitised so they were able to say, yes, I can cooperate, in what ever form. In this respect, the round tables did have a key function in the implementation process, I guess you can say that. |
| #7 | “eigentlich hätte man das so machen müssen, aus dem Bereich der Gebietskooperation Hase einen Unterhaltungsverband, einen Kommunalvertreter, einen Landkreisvertreter, ein Forstvertreter, einen Fischereivertreter und einer der Naturschutzverbände. Das wäre sechs Leute plus ein bisschen Verwaltung, dann wären Sie bei 10 Leuten gelandet und das waren nachher weit über 20. So, weil, weil die Kommunen und die Landkreise - jeder wollte dabei sein, weil sonst könnte ja etwas zu seinem eigenen Nachteil da beschlossen werden. Und das waren schon mit die größten Startschwierigkeiten. Wer kommt überhaupt mit in diesen, in diesen Kreis rein und irgendwelche Angler sind dann an den Umweltminister rangegangen und dann wurde per Erlass beschlossen, dass die Fischereileute auch noch mit sitzen dürfen mit 2 Plätzen. Also einmal den behördlichen Fischereischutz und den […] ehrenamtlichen Fischereischutz! Und da muss man die Frage stellen, das hätten wir mit Sicherheit kleiner aufziehen müssen. Und dann auch stringent. Dann wäre man glaub ich bei der ein oder anderen Fragestellung da besser bei gefahren. Es ist einfach so, wenn sie Gruppen haben über 20 Leute, [die] sind schwer steuerbar.” (Maintenance association, #19:38#) | Actually, for there realm of the area cooperation Hase, one should have nominated one person from the maintenance association, one representative from the municipalities, one representative from the countries, one from forestry, one representative from fisheries and one from the nature conservation organisations. This would have been six people, plus some from the administration which would have yielded about 10 people. In the end, there were far more than 20 people, because everybody from the municipalities and the counties wanted to participate in case something would be decided to one’s disadvantage. This was one of the biggest challenges in the beginning. Who is allowed to be part of this round? Some anglers went to the Environment Minister and eventually it was decided per decree, that people of the fisheries would also get two seats. One for the governmental and one for the voluntary fishery organisations. And you would have to ask, we would have to organise this in a smaller way. And more stringent. I think it would have been better for one or the other issue that arose. It’s just difficult to have groups with more than 20 people, they are hard to steer. |
| #8 | “Und der Anspruch war ja auch da, dass die Gebietskooperationen stringenter organisiert sind, da sollte eben ein Vertreter der Städte und Gemeinden, […] einer vom Landkreis [sein], auch wenn zwei oder drei Landkreise beteiligt waren. Aber es wurden meistens alle die kamen da mit aufgenommen, und dadurch wurde es immer mehr zum Lauschclub als zum Diskutierclub.” (environmental organisation, #13:56#) | And the idea was also that the area cooperations should be organised in a more stringent way, that there should be one representative from the cities and municipalities, […] one from the county, even if one or two counties were involved. But most of the time, everybody that came was included and this way, there was less of a discussion and more of people merely listening. |
| #9 | „Weil wir hatten ja alle Naturschutzverbände; in Niedersachsen haben wir 13 anerkannte Verbände, da gehören auch die Fischereileute dazu, da gehören auch die Jäger dazu, und was weiß ich was dazu gehört, irgendwelche Wandervereine. Aber die großen beiden sind natürlich der NABU und der BUND. [...] inzwischen wohl hatte auch diese Aufteilung der Flussgebiete in Teileinzugsgebiete [stattgefunden, das] hatte damals noch NLÖ [Niedersächsisches Landesamt für Ökologie] gemacht. Dann haben wir gesagt, für jedes Teileinzugsgebiet werden wir dann ohne weiteres einen finden, aber das war nicht so leicht. Letztendlich haben wir dann für jeden einen gefunden, aber erst so nach 1 - 1,5 Jahren.“ (Environmental organisation, #09:54#) | Because we did have all of the nature conservation organisations: in Lower Saxony we have 13 accredited organisations, which include fisheries, huntsmen and whatever else, some hiking clubs. But the biggest two are of course NABU and BUND. In the meantime, the delineation of the river basins in sub-basins was done by the NLÖ [state agency for ecology]. And then we said that we would easily find one representative for each sub-basin but it wasn’t so easy. Essentially we did find one for each sub-basin but only after 1 to 1 ½ years. |
| #10 | “Die Gewässer haben auch bestimmte Aufgaben - ganz klar - und wenn man sich dieses Gebiet hier anschaut - das ist landwirtschaftlich und infrastrukturell sehr stark geprägt und wenn man diese Nutzung und Infrastruktur aufrecht erhalten will, müssen Gewässer eben bestimmte Funktionen erfüllen. So und ich denke mal, die Angst die da damals mit bei den Unterhaltungsverbänden vorherrschte ist mit Sicherheit gewesen, dass diese, diese Funktion der Gewässer nicht mehr vorhanden sein könnten mit den entsprechenden negativen Folgen. Man kann nicht [für] Gewässer einfach die Räumung einstellen. Das geht nicht. Das hat negative Auswirkungen auf die Infrastruktur - landwirtschaftliche Flächen, bebaute Bereiche, aber auch auf das Pflanzen und Artenpotential in diesen Gewässern.“ (maintenance association, #08:25#)  „Also ich sage jetzt mal so, wenn ich jetzt eine Flurneuordnung starte, und da muss jetzt der Straßenbau, der da stattfindet, der muss kompensiert werden, dann versucht man das natürlich schon in diesen Regionen mit den Bächen schon so hinzukriegen, dass das natürlich auch da in das Konzept hineinpasst. Also sprich Uferbepflanzung und was auch immer. Das ist das eine. Das andere ist natürlich, bei allem was man tut, sollte man natürlich auch das Ende bedenken. [,...] das sehe ich dann so oft, das wird immer gerne gefordert, die kompletten Bäche sollen bepflanzt werden, möglichst von beiden Seiten, dann ist die Unterhaltung dieser Bäche natürlich wieder schwierig. “ (agricultural association, #43:27#) | The water bodies clearly have determined functions. And if you look at this area, which is characterized by intensive agriculture and highly developed infrastructure, if you want to keep this utilisation and infrastructure, the water bodies have to fulfil certain functions. And I think the fear that most of the maintenance associations surely had was that the function of the water bodies wouldn’t exist anymore, which would have negative consequences. You cannot just stop cleaning and clearing water bodies. It doesn’t work that way. It has negative consequences for the infrastructure, agricultural areas, cultivated areas but also on the potential of plants and species in these water bodies.  I am phrasing it this way, if I am starting a rezoning programme and the road construction that is happening there has to be compensated for, you do try to implement it in the areas with water bodies in a way that fits into the concept. Meaning plantation of the banks and whatever. This is one thing. The other thing is of course, in everything you do, you should keep the end in mind. I see that a lot, people demanding that all of the creeks should be vegetated, preferably from both sides, that makes the maintenance of these creeks more difficult of course. |
| #11 | “Also zum Beispiel die Stadt [anonymisiert] bearbeitet das Thema Gebietskooperation, oder Wasserrahmenrichtlinie, auch viel mehr im Sinne von Natur erleben, oder Öffnung der Gewässer für die Menschen, als dass sie sich die Qualitätskomponente Makrozoobenthos, oder Fische; das tut sie natürlich auch, aber immer natürlich im Kontext ‘schön, dass da Fische drin sind, weil das ist ja dann auch ein lebenswerter Lebensraum für die Menschen drum herum.’” (county, #12:47#)  „Der Stand der Umsetzung, und was man noch machen kann. Wo man noch irgendwelche Sachen anpacken kann. Wir haben hier auch noch den Verein zur Revitalisierung der Haseauen, die auch viel an Umsetzungsarbeiten am oder im Gewässer machen. Das meiste ist zwar *um* den Gewässern, weil da dann die Zweckverbände alle schon wieder reinspielen, die lieber den Tourismus fördern wollen als die Umwelt. Deshalb haben wir teilweise hier auch viel mit Kanufahrern zu tun jetzt auf der Hase. Und der dann auch noch immer mehr gefördert wird, weil es da ja wieder Gelder gibt.” (environmental organisation, #45:05#) | For example the city of […] looks at the issue of area cooperation or Water Framework Directive more from a perspective of making the water bodies more accessible for the people, providing a better access to nature instead of looking at the quality component of fish or macrozoobenthos. They do look at that but they evaluate it more in sense of “it’s a good thing that there are a lot of fish because that makes it a more attractive environment for the people”.  The status of the implementation and what else could be done. Where other measures can be implemented. We have another association whose purpose is the revitalization of the Hase wetlands. They did a lot of implementation of measures around the water bodies. Most of it is done *around* the water bodies because the maintenance association would rather promote tourism instead of the environment. This is why we have to deal quite a lot with canoeists on the Hase. And there is quite a big promotion of that because there are more funds available. |
| #12 | “im Gesamteindruck fand ich eher, dass das ein Ideenpool war teilweise und man auf der Basis eher relativ konstruktiv zusammen gearbeitet hat. Also ich hatte jetzt nicht dein Eindruck, dass da ganz große Gegensätze aufgetaucht hat. Selbst die, man müsste ja jetzt eigentlich denken, dass die Landwirte, die Vertreter der Landwirtschaft eher da, jetzt gegen gearbeitet haben, das stimmt aber nicht. Im Endeffekt sind, haben wir akzeptiert, dass die Gesetzeslage so ist in einigen Bereichen und man hat halt diskutiert wie man die Probleme angehen kann.“ (water treatment, #30:46#) | Regarding the overall impression, I would say that it was a pool of ideas and a rather constructive cooperation. I didn’t have the impression that there were many differences. Even then, you would think that farmers and representatives of agriculture would work against this but that wasn’t true. In the end, we accepted that the legal situation is the way it is in some areas and we were discussing how to solve the problems. |
| #13 | “Das fehlende Geld war eigentlich immer ein Dauerthema und die schon erwähnten 15.000 EUR waren ja im Grunde genommen verhinderten sie dass man wirklich intensiv plante, weil man genau gewusst hat, es bringt doch nichts dass man irgendwelche Überlegungen anstellt. Wir wissen dass das Land Niedersachsen nicht mehr als diese 15.000 EUR zur Verfügung stellt“ (Maintenance association, #17:56#)  “Die große Problematik ist die Finanzierung. Das ist einmal das ganz große Problem, was wir haben. Und da hat man sich offensichtlich auch, als man diese Wasserrahmenrichtlinie da vollmundig, ich sage mal, propagiert hat, und reingetragen hat ins Land, da hat man sich wahrscheinlich im ersten Moment noch keine Gedanken darüber gemacht: Was kostet das eigentlich?” (agriculture, #4:30#) | The lacking funds were a permanent issue and the aforementioned 15,000 EUR essentially prevented that anyone did intensive planning because you knew, that making plans didn’t make sense. We know that the federal state of Lower Saxony won’t provide more than these 15,000 EUR.  The biggest problem is the funding. This is one of the main problems that we are facing. And clearly this wasn’t thought of when the Water Framework Directive was introduced and propagated: How much is this going to cost? |
| #14 | “Ich denke mal schon, dass es mit Sicherheit zu einer Annäherung der einzelnen Positionen über die Jahre geführt hat und auch zu einer erhöhten Akzeptanz gegenüber den Denkansätzen der anderen Teilnehmer. Das muss man glaub ich schon sagen“ (maintenance association, #29:25#)  „für uns wars wirklich interessant und für uns hat - sind das Informationen, die wichtig sind um auch die Einleitung beurteilen zu können und um gegebenenfalls auch darauf hin zu wirken, dass man gegebenenfalls die Abwasserbeseitigungsstruktur vielleicht auch ändert. Wir haben zum Beispiel jetzt äh, ja wir wissen ja, dass der Mühlenbach zum Beispiel in Berge ein prioritäres Gewässer ist, ein FFH Gewässer [unter der EU Flora-Fauna-Habitat Richtlinie] und wir haben jetzt die Kläranlage in Berge aufgehoben nach Nortrup umgeleitet, das bot sich an, wobei der Waldbach in Nortrup ebenfalls ein FFH Gewässer ist, aber so können wir praktisch ein FFH Gewässer schonen.“ (Water treatment, #11:39#)  „natürlich wenn es ums Thema Grundwasser ging bin nicht nur ich Akteur, sondern sind auch die unteren Wasserbehörden Akteur und auch verschiedene Kommunen und auch die Landwirtschaftskammer [...] hat auch entsprechende Fachkenntnisse, die sie einbringen kann. Deswegen wurde da schon sich fachlich gut ausgetauscht und es ist aus meiner Sicht eben auch eine Bereicherung und ein Erkenntniszugewinn durch diesen Austausch. Also man, man hat natürlich [...] festgestellt, dass man [... über die] Betroffenheit der anderen viel besser Bescheid wusste und das anders einschätzen konnte.“ (Water treatment, #19:18#) | I would think that it surely led to a harmonisation of the individual positions over time and also to a bigger acceptance of other participants’ approaches. I think one could say that.  It was really interesting for us and these are pieces of information that are important in order to evaluate the discharge and if necessary work towards changing the structure of sewage disposal. For example, we know that the Mühlenbach in Berge is a water body that has high priority, a FFH water body [under the EU Habitat Directive] and we did transfer the sewage treatment plant to Nortrup even though the creek in Norttrup is also a FFH water body. But this way we were able to conserve one FFH water body.  When we were talking about groundwater I am not the only stakeholder, there are the lower water authorities, the different municipalities and also the chamber of agriculture, which has corresponding expert knowledge that they could introduce. Therefore, there was a good professional exchange and in my point of view, there was an enrichment and also an acquisition of new knowledge and awareness due to this exchange. I noticed that it got easier to know about and estimate the other stakeholders’ concerns. |
| #15 | “Es war so, dass wir, dass es eine kurze Zeit gab, wo wir Maßnahmen zu benennen hatten, die wir auch in diese drei Schubladen eingepackt haben, was im Grunde auch ein Stück weit hier bei uns im Hase-Gebiet entwickelt wurde, weil man einfach Angst hatte vor der Verantwortung, dass man nachher für die Finanzierung verpflichtet wird. Das ist hier sicherlich ein bisschen intensiver diskutiert worden, insgesamt hat es hier eine Maßnahmenliste gegeben mit dieser großen Anzahl von diesen Maßnahmen, die dort benannt wurden, aber die Maßnahmen wurden im Prinzip ausschließlich von Unterhaltungsverbänden benannt. [...] ansonsten wars tatsächlich schon so, dass das von den Unterhaltungsverbänden geprägt war und dann nurmehr die Ergebnisse in der großen Runde in der Kooperation vorgestellt wurden. So das was wir auf Papier gebracht haben, das wurde an die Wand geschmissen, konnte auch jeder sich downloaden im Wasserblick [deutsche WFD online Plattform], aber dort haben sich die wenigsten noch intensiver eingebracht.” (NLWKN, #01:02:53#) | There was a short time, in which we had to denominate measures, which we did assign to one of the three categories. This was developed partly in our area, the Hase-area because there was this fear of the responsibility, that one would be obligated to funding it. This surely was discussed more intensely here. Overall, there was a list of measures with this big amount of measures but the measures were basically exclusively denominated by the maintenance association. […] most of the time things were determined by the maintenance organisation and then the results were presented in a big round. So what we developed was presented and everybody could download it in the “Wasserblick” [German WFD online platform] but this was were not many people were participating intensely. |
| #16 | “Und das Entscheide war dann, es gab ja die Ausweisung der erheblich veränderten Gewässer, heavily modified waterbodies, da hatten die NLWKN, [...] weiß ich nicht, die das zuerst bearbeitet haben. Die hatten einen Vorschlag gemacht, wie viel Prozent ausgewiesen werden als heavily modified, und wie viel nicht. Und dann hat die Landwirtschaft erkannt: Wenn wir Gewässer alle als erheblich verändert ausweisen, dann müssen wir nicht so viel an den Gewässern machen. [...] daher sind wir in Niedersachsen bei 85%.“ (Environmental organisation, #28:36#)  „Und es war so, das war die einzige Aufgabe, wo die Gebietskooperationen selber was gemacht haben. Die sollten die erheblich veränderten Gewässer ausweisen.“ (Environmental organisation, #30:20#) | The important thing was, there was the denomination of the heavily modified waterbodies, I think it was the NLWKN […], or I don’t know, that first worked on this. They made a proposal regarding what percentage would be identified as heavily modified and what percentage wouldn’t. And then the agriculture realized, if we identified all waterbodies as heavily modified, then we wouldn’t have to do as much […] and this is why we are at 85% in Lower Saxony.  This was the only task that the area cooperations did themselves. They had to identify the heavily modified water bodies. |
| #17 | “Aber es gab auch früher schon mal die Möglichkeit, dass zum Beispiel wenn ein Absturz umgebaut werden sollte in eine Sohlgleite, wurde ein Förderantrag gestellt und dann hat der Unterhaltungsverband zum Beispiel gesagt, das Ding bauen wir für 15.000 EUR. Wir machen das mit eigenem Gerät, mit eigenem Personal, erhöhen dadurch die eigene Wirtschaftlichkeit und das Land spart Geld dadurch. Aber mit ja, FDP Politikern im Land wurde gesagt, das wollen wir nicht mehr, es muss alles ausgeschrieben werden und es muss vergeben werden. So und dadurch ist, so seh ich das, und auch ein paar andere Kollegen, gewisses Interesse an solchen Maßnahmen-na klar-erlahmt.“ (Maintenance association, #46:18#) | There has been a previous possibility, for example to rebuild a drop into a river bank revetment, an application for support was submitted and the maintenance unions said, in order to do this we need 15,000 EUR. We are doing it with our own equipment, our own staff, we are increasing the cost effectiveness and the [federal] state is saving money. But yeah, with politicians from the FDP [German liberal party] it was said that this doesn’t work that way anymore, everything has to be tendered and then awarded to someone. And in my and also some colleagues’ point of view, this led to a decrease of interest in these kind of measures. |
| #18 | “[…] darauf haben wir ja auch gesehen, dass nicht mehr alles Holz weggemacht wird. Und wir werden jetzt sehen, wie es sich hier weiterverhält. Und somit sind wir hier in diesen Gebiet eigentlich Vorreiter, wieder Bäume am Gewässer stehen zu lassen, um auch eine Beschattung in den Gewässern zu kriegen, und ein Ufererhalt. Und dass man dort einen vernünftigen Fluss wieder hat.“ (Fishery, #14:40#) | […] regarding that we also noticed that not all of the wood was removed. We will now see how this will go on. This makes us pioneers in this area, to have more trees at the water bodies in order to get shadows and a preservation of banks into the water bodies. In order to create a better stream. |
| #19 | “also es war so, dass natürlich auch die Ziele der Wassrrahmenrichtlinie den Unterhaltungsverbänden bekannt waren und die waren natürlich bemüht im Rahmen ihrer Unterhaltungsmaßnahmen auch Querbauwerke zu entfernen. Das ist ja dann auch so nach und nach erfolgt, [...]. Von Hammerstein haben sie an der Mühle haben sie auch ein Querbauwerk wieder zusammen entfernt und ja, das sind alles so Dinge, die werden ja nicht vom Land unterstützt, sondern die laufen praktisch dann auf der Ebene der Unterhaltungsverbände was jetzt Oberflächengewässer betrifft. Aber so direkt, dass die Gebietskooperation irgendwelche Maßnahmen umgesetzt hat, die dann jetzt wirklich dann auch eine maßgebliche Aufgabe - also sagen wir mal eine maßgebliche Erfüllung der Wasserrahmenrichtlinie herbeigeführt hätten [...] - das kann man nicht sagen. Das, weil einfach da der - ja wie soll ich sagen - im Endeffekt wären die, sind die Kosten für die Umsetzung der Wasserrahmenrichtlinie viel zu hoch. Das muss über Fördermittel durchs Land erfolgen, langfristig. Zum Beispiel hat man jetzt über Flurbereinigung - ja, da hat der Landkreis das ein oder andere renaturiert. Zum Beispiel am Reitbach sind glaub ich mittlerweile 2-3km mit einer Aue versehen worden im Zuge von Flurbereinigungsmaßnahmen. Das sind dann alles so Maßnahmen wo man versucht Ausgleichsflächen zu, ja einzubinden, um letztlich auch die Ziele der Wasserrahmenrichtlinie umsetzen zu können. Wir haben auch bei dem Ausbau der Kläranlage in Nortrup mussten wir ja auch Ausgleich schaffen und da haben wir auch einen Teil dieser Strecke am Reitbach mitfinanziert. Ja, 10.000 EUR oder sowas. Und das ist auch Sinn der Gebietskooperation, dass man letztlich auch solche Sachen, ja, gezielt zur Erfüllung der Ziele der Wasserrahmenrichtlinie nutzt. Ja, und das wird im Prinzip durch diese Gebietskooperation auch mit forciert. Das ist ein ganz wichtiger Ausfluss der Gebietskooperation.“ (treatment association, 2-#04:26#) | The maintenance organisations of course knew about the goals of the Water Framework Directive and they made an effort within the realm of their measures of maintenance to remove barriers. This did happen little by little. They did remove structural obstacles starting from Hammerstein at the mill so these are all things that are not supported by the [federal] state but are more happening on the level of the maintenance associations, regarding surface waters. But in a sense that the area cooperation implemented measures that significantly led to a compliance with the Water Framework Directive – I don’t think this happened. Because in the end, the costs for the implementation of the Water Framework Directive are way too high. In the long run, this would have to be done through subsidies of the [federal] state. There were some renaturations by the county, for example the Reitbach, I think there are now 2-3 km of wetlands as part of plot realignment. These are all types of measures where you try to include buffer strips in order to achieve the goals of the Water Framework Directive. As part of the extension of the sewage treatment plant in Nortrup we also had to create buffer strips and we simultaneously funded part of the works at the Reitbach. I think it was something like 10,000 EUR. The use of the area cooperation is ultimately that you use things like that in order to achieve the goals of the Water Framework Directive. And essentially it is also enforced through the area cooperation. It’s a very important part of the area cooperation. |
| #20 | “Q: Um es da vielleicht ein bisschen umzubrechen, was wären dann so die Interessen der Kommunen? […]  A: Also ein Aufhänger für die Gemeinden ist sicherlich bei manchen Hochwasserschutz gewesen. Dann ist es das Landschaftsbild. Für die Gewässer dritter Ordnung habe ich ganz klar die rechtliche Aufgabe der Gewässerunterhaltung, sprich der Pflege und Entwicklung.” (municipal association, #17:20#)  „Q: Sie haben gerade gesagt „typische gemeindliche Interessen“. Was sind das? Was ist, in dieser Gebietskooperation, die da reingetragen worden?  A: Ja, typische gemeindliche Interessen, das sind diejenigen Fragen, mit denen wir uns täglich auseinandersetzen. Bauleitplanung, also die über Bauleitplanung umgesetzt werden. Fragen der Attraktivität der Landschaft, Erholungsnutzen von Landschaft. Aber auch die ganzen Interessen, wenn man all die Verantwortlichkeiten, insbesondere existenzielle Fragen wie Hochwasserschutz nimmt, dann ist das natürlich ein Interesse der Gemeinde. Wo man natürlich auch gerade beim Thema, wo es um das Wasser geht, wo man als Gemeinde auch froh ist, wenn man sagen kann: Das, was passiert, passiert auch im Interesse des Hochwasserschutzes, für den wir ja selbstverständlich verantwortlich sind.“ (municipality, #36:40#) | Q: To break it down a bit more, what were the interests of the municipalities? […]  A: For some municipalities flood protection was certainly one access point, and the scenic value of the landscape. For third order water bodies, they clearly have the legal responsibility of maintenance and development.  Q: You just mentioned ‘municipal interests’. What are these? What was brought to the table in this area cooperation?  A: Typical municipal interests are those issues that are occupying also our every-day work. Things that are implemented through land use planning. Issues of the scenic and recreational value of the landscape. But also, regarding responsibilities, existential issues such as flood protection. When it comes to issues of water, you are glad as a municipality when you can say: the things that are done are also in the interest of flood protection, for which we are responsible. |
| #21 | “Q: War die Größe der Gebietskooperationen, eben orientiert an diesen Teileinzugsgebieten, war das sinnvoll?  A: Wie ich sagte, Schleswig-Holstein hat genauso so viele Arbeitsgruppen wie wir Gebietskooperationen haben. Während Niedersachsen doppelt so groß ist. Also die sind schon ziemlich groß. [...] Man könnte sagen: Grenzwertig. [...] und das Problem natürlich, und da muss man sich auch klar darüber sein, dass als Naturschutzverband, auch der Leuchtturm [deren Repräsentant] oder wie auch immer, der wird auch nicht alles kennen.“ (Environmental organisation, #36:32#) | Q: Was the size of the area cooperation oriented at the sub-basins, did that make sense?  A: As I said, Schleswig-Holstein [neighboring federal state] has as many working groups as we have area cooperations. Lower Saxony is twice as big so you could say that they are pretty big. […] you could say borderline […] and the problem which one has to keep in mind is that the nature conservation organization and his representative is not going to know everything. |
| #22 | “Aber bei einer letzten Besprechung sind aus den Maßnahmenplänen diese Kooperationsgruppen entwickelt wurden. Da ist ja zum Beispiel eine für den Morsbach, und da bin ich zuletzt mit beigewesen, und da wurde dann auch in Gruppen vor den Plänen diskutiert. Das heißt also, da das [Planungsbüro], das Vertreter zu den Gesprächen gestellt hatte, die Pläne überall ausgehängt hatte und man konnte dann vor den Plänen mit diesen Vertretern des Planungsbüros die Dinge besprechen, das fand ich eigentlich sehr gut. Man konnte sich die Pläne ansehen und hatte dann vielleicht die lokalen Kenntnisse.” (Nature protection, #12:58#) | In one of the last meetings the programme of measures led to the development of the cooperation groups. For example, there is one for the Morsbach in which I was involved until the end and this is where the groups in front of these plans and maps. This means, the planning bureau sent representatives to the meetings and distributed the plans everywhere and this way, you could discuss the plans with representatives of the planning bureau, which I thought was a good thing. You could look at the plans and then maybe held the local knowledge. |
| #23 | “Zur WRRL bin ich gekommen als Vertreter des [anonymisiert], und in diese Schiene kamen die Einladungen zum jeweils Runden Tischen - das ist ja eine Vielzahl mittlerweile, das kann ja ein Ehrenamt fast gar nicht mehr leisten.” (local agricultural association, #02:07#) | I came to the WRRL as a representative of [anonymised] and this is how we got the invitations to each round tables. There are so many by now, it is almost impossible to do it as a volunteer. |
| #24 | „diesen Dachverband gibt es noch gar nicht so lange. Rund um die Hase gibt es einige Institutionen, die irgendwann mal gegründet worden sind, die auch teilweise schon sehr alt sind. Aber dieser Dachverband, also dieses Dach über den betroffenen Unterhaltungsverbänden ist wirklich zu Beginn der Wasserrahmenrichtlinie gegründet worden. Hatte am Anfang noch gar nicht so viel, böse gesagt, dämmerte ein bisschen vor sich hin, oder hat man erst mal nicht richtig wahrgenommen in seiner Funktion. Das hat sich geändert, seit dem es wirklich Maßnahmen gibt, wo eben dann auch die Trägerschaft ein Thema wurde. Und dann der Dachverband als Antragsteller aufgetaucht ist im Rahmen der Förderrichtlinie.“ (county 2-#1:16#) | This umbrella organisation hasn’t been in place for so long. There are some institutions in the Hase area that are quite old but this umbrella organisation of maintenance organisations was founded right in the beginning of the Water Framework Directive. In the beginning it didn’t do that much, its function hasn’t really been obvious. But ever since measures were introduced this has changed, when the sponsorship and responsibility became a bigger topic. This is when the umbrella organisation emerged in the context of the directive. |
